# S100-Alarmins Are Essential Pilots of Postnatal Innate Immune Adaptation

**DOI:** 10.3389/fimmu.2020.00688

**Published:** 2020-04-30

**Authors:** Dorothee Viemann

**Affiliations:** ^1^Department of Pediatric Pneumology, Allergy and Neonatology, Hannover Medical School, Hanover, Germany; ^2^Cluster of Excellence RESIST (EXC 2155), Hannover Medical School, Hanover, Germany; ^3^PRIMAL Consortium, Hanover, Germany

**Keywords:** neonate, S100A8/A9, systemic immunity, innate immunity, trained immunity, immune adaptation, inflammatory diseases

## Abstract

The restricted capacity of newborn infants to mount inflammatory responses toward microbial challenges has traditionally been linked to the high risk of septic diseases during the neonatal period. In recent years, substantial evidence has been provided that this characteristic of the neonatal immune system is actually a meaningful physiologic state that is based on specific transiently active cellular and molecular mechanisms and required for a favorable course of postnatal immune adaptation. The identification of physiologically high amounts of S100-alarmins in neonates has been one of the crucial pieces in the puzzle that contributed to the change of concept. In this context, innate immune immaturity could be redefined and assigned to the epigenetic silence of adult-like cell-autonomous regulation at the beginning of life. S100-alarmins represent an alternative age-specific mechanism of immune regulation that protects neonates from hyperinflammatory immune responses. Here, we summarize how infants are provided with S100-alarmins and why these allow an uneventful clash between the innate immune system and the extrauterine world. The mode of action of S100-alarmins is highlighted including their tuning functions at multiple levels for establishing a state of homeostasis with the environment in the newborn individual.

## Introduction

After transition from the largely sterile intrauterine environment, the immune system encounters a flood of antigenic stimuli in the new extrauterine world. This represents an enormous challenge for the newborn infant and initiates a process of immune adaptation. Ideally, early-life immune adaptation is a training by new antigens that goes along with the acquisition of protecting immune memory. Especially during the neonatal window of life, responses to new antigens have lifelong imprinting effects by transcriptional and epigenetic reprogramming of systemic and mucosal immunity (1–8). Therefore, early-life immune adaptation critically determines the long-term development of health and disease. Successful immune adaptation achieves an energy-efficient state of homeostasis which balances tolerizing and defending activities of the immune system toward the environment. If immune adaptation fails, the activity of the immune system remains unbalanced which sooner or later leads to the development of chronic inflammatory diseases and an increased susceptibility to infections (2, 3, 5, 9–14). With respect to systemic immunity, important direct reprogramming immune challenges are commensal microbes colonizing the host's barrier sites including thereof translocating metabolites, infections, vaccines, and metabolized food components (2, 3, 5, 7). Therefore, in terms of promotion of successful immune adaptation, a myriad of excellent work has been done and still goes into identifying the ideal pattern of developing microbiota compositions, dissecting “bad” and “good” early-life infections, testing optimized kinds, and combinations of vaccines and defining the most favorable diet including probiotics for infants and young children. On the other side, the mechanisms and molecular options, the newborn infant has to impact on the process of immune adaptation, are still barely elucidated. The interindividual differences in coping with the flood of new antigens after birth and the postnatal development of immunity indicate that host factors must play an important role for the outcome of immune adaptation. Host factors might exert direct imprinting effects and/or determine how the neonate meets its new environment by regulating the neonatal immune response. In this respect, the alarmins S100A8 and S100A9 have been identified as important host factors warranting uneventful and favorable postnatal immune adaptation. In this review, we highlight the current knowledge about the sources of S100A8/A9 supply to the newborn infant and the mode of action of S100A8/A9 in controlling postnatal innate immune adaptation.

## Protein Biochemistry and Signaling of S100A8/A9

S100A8 (also named calgranulin A; myeloid-related protein 8, MRP8) and S100A9 (calgranulin B; MRP14) are two members of the S100 protein family specifically linked to innate immune functions. They are calcium-binding proteins characterized by two calcium binding EF-hand motifs, which are connected by a central hinge region. Physiologically, S100A8 and S100A9 exist only as heterodimer S100A8/A9 (termed calprotectin) that can form heterotetramers in the presence of calcium or zinc, but not or at most very little as S100A8/A8 or S100A9/A9 homodimers (15, 16). The reason for it is the instability of the homodimers. Two independent laboratories have shown that myeloid cells of the *S100a9*-knockout mouse express *S100a8* mRNA but no S100a8 protein, suggesting that the posttranscriptional *in vivo* stability of S100a8 protein is dependent on S100A9 expression (17, 18). Interestingly, the deletion of the mouse *S100a8* gene results in an embryonically lethal phenotype without detectability of S100A9 homodimers, pointing to an important role of S100A8 during embryogenesis (19). Once released to the extracellular space, S100A8/A9 impacts on immune cells through binding to different cell surface receptors.

In inflammatory disease states, S100A8/A9 has been shown to bind to endothelial cells (ECs) and chondrocytes by a mechanism involving heparan sulfate proteoglycans and carboxylated N-glycans (20–22). The multiligand receptor for advanced glycation end products (RAGE) was also proposed to act as an S100A8/A9 receptor (23, 24). However, in healthy conditions, the physiological relevance is debatable as RAGE is widespread (on vascular ECs, neutrophils, monocytes/macrophages, lymphocytes, dendritic cells, cardiomyocytes, and neurons) but relatively low expressed (25). Moreover, the binding of S100A8/A9 to RAGE is rather weak and was shown to rely on S100A9 and the presence of Ca^2+^ or Zn^2+^ ions (26). Therefore, it is still controversial and questionable that RAGE mediates S100A8/A9 signal transduction.

The main signaling pathway of S100A8/A9 has been delineated by Vogl et al. who demonstrated that S100A8/A9 is a ligand of the Toll-like receptor 4 (TLR4) (27–30). A direct binding of S100A8 and S100A9 to the TLR4–myeloid differentiation factor 2 (MD2) complex was confirmed by surface plasmon resonance studies (29) and comprehensive binding assays (30). The discovery of S100A8/A9 as endogenous TLR4 ligand has been groundbreaking as before TLR4 was only known as a pattern recognition receptor (PRR) for the exogenous TLR4 ligand lipopolysaccharide (LPS), the integral part of the outer membrane of gram-negative bacteria. Fueled by this work, the concept of endogenous damage-associated molecular pattern molecules (DAMPs, also termed alarmins) evolved in analogy to the exogenous microbe-associated respective pathogen-associated pattern molecules (MAMPs respective PAMPs) as activators of PRRs. The canonical downstream signaling induced by TLR4 ligation through S100A8/A9 and LPS has a huge overlap (27–29). Similar to LPS (31, 32), the binding of S100A8/A9 induces the translocation of the adaptor molecule MyD88 from the cytosol to the TLR4 receptor complex which subsequently activates interleukin-1 receptor–associated kinase-1 (IRAK-1) and the transcription factor nuclear factor (NF)-κB p65/p50, thereby increasing the expression of a classical pro-inflammatory gene program including, e.g., tumor necrosis factor (*TNF*), interleukin (*IL*)*6, IL1B*, C-X-C motif chemokine ligand 2 (*CXCL2*), and C-C motif chemokine ligand 2 (*CCL2*) (8, 27–30). However, in contrast to LPS, at least in neonatal monocytes, it was shown that S100A8/A9 does not activate the TLR4 adaptor molecule TIR domain-containing adaptor protein-inducing interferon-β (TRIF)-TRIF-related adaptor molecule (TRAM) (8). The reason for it has not been completely elucidated yet, but S100A8/A9 seems to bind different domains of the TLR4/MD2 receptor complex than LPS (29, 30). TRIF-dependent TLR4 signaling shifts the transcription factors IFN regulatory factor (IRF)3 and IRF7 into the nucleus and activates a regulatory gene program including, e.g., *CCL5, CXCL9, CXCL10, CD80*, and IFN-response genes that in turn regulates the LPS response in a cell-autonomous manner and also links the innate to the adaptive immune system (33–36).

**Figure 1:**
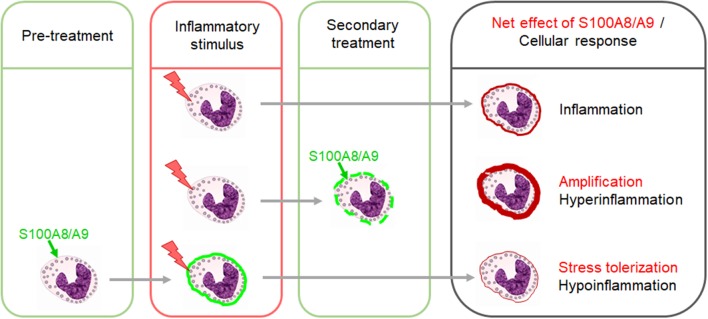
Dual impact of S100A8/A9 on the outcome of inflammatory responses. The secondary release of S100A8/A9 during an inflammatory response upon a preceding stimulus has amplifying effects. Excessive S100A8/A9 release in such settings increases the risk of hyperinflammation. In contrast, pretreatment of immune cells with S100A8/A9 induces a state of hyporesponsiveness of innate signaling pathways which dampens the response to subsequent inflammatory stimuli.

When released secondarily during a primary acute inflammatory process like sepsis and infection or autoimmune and autoinflammatory reactions, S100A8/A9 amplifies the primary pro-inflammatory response by activating the MyD88-dependent TLR4 signaling pathway (29, 37–42) (Figure 1). In adults, the sepsis amplifying effect was proposed to be pathogenetically relevant. In a small study in 17 septic shock patients, S100A8/A9 levels decreased in surviving patients during recovery while non-survivors were characterized by high S100A8/A9 serum levels (43). This could be validated in another cohort of 49 septic shock patients with high plasma levels of S100A8/A9 being associated with a higher risk of death (42). However, under sterile stress conditions with no underlying primary inflammatory stimulus, S100A8/A9 does not induce an inflammatory disease state but contrary a state of inflammatory hyporesponsiveness to subsequent inflammatory stimuli, particularly microbial challenges (Figure 1) (8, 27, 44). However, S100A8/A9 was shown to tolerize not only for a subsequent TLR4 signaling but also TLR2 signaling (27). This work led to the introduction of the concept of “stress tolerance” by endogenous DAMPs and completed the proof of principle that DAMPs similar as PAMPs are able to act in a dual manner, i.e., pro-inflammatory when released secondary and regulatory under sterile conditions. No matter whether pro-inflammatory or regulatory, the S100a8 homodimer proved in several studies as much more potent than the S100a9 homodimer or the heterodimer S100A8/A9 (8, 27, 45). The question of the reason for it could be solved recently. S100A8 and S100A9 bind to the TLR4 complex *via* specific peptide sequences (29, 30). It was shown that calcium-induced (S100A8/A9)_2_ tetramer formation hides the TLR4-binding site and blocks the ability of the heterodimer to further bind to TLR4 which prevents undesirable systemic effects (30). This work provided the explanation for how S100A8/A9 effects are locally restricted in sterile settings.

An important molecular mechanism of stress tolerance induction is the S100A8/A9-TLR4-MyD88-mediated preactivation of NF-κB. After activation, cytosolic NF-κB shifts into the nucleus and is then subjected to rapid proteasomal degradation (8, 46, 47). Thus, after S100A8/A9-conditioning, cytosolic NF-κB is no longer available for the canonical signaling cascades of subsequently activated innate signaling pathways which except for TLR3 all depend essentially on NF-κB (31). Yet, recently, it could be revealed that S100A8/A9 priming induces still more in-depth reprogramming of immune cells than only “NF-κB consumption.” In human as well as murine monocytes/macrophages, two major pathways responsible for the S100A8/A9-primed hyporesponsiveness could be identified (44). Firstly, S100A8/A9 induced a lasting inactivation of the phosphatidylinositol 3-kinase (PI3K)/AKT/GSK-3β pathway which resulted in an accumulation of NF-κB inhibitors. Secondly, IL-10-dependent STAT3 activation and nuclear BCL-3 accumulation were identified as master regulators of S100A8/A9-induced tolerance, with the latter resulting in an inhibition of NF-κB transactivation.

## Systemic Supply of the Newborn Infant With S100A8/A9

In health, significant expression of S100A8 and S100A9 has been found only in cells of myeloid origin, i.e., granulocytes, monocytes, and macrophages (48, 49). S100A8 and S100A9 constitute up to 40% of cytosolic proteins in granulocytes and 5% in monocytes (50). Inducible expression in microvascular ECs has been described (51, 52) in experimental lung cancer but not under physiologic conditions (51). In psoriasis and other inflammatory skin diseases, S100A8 and S100A9 are highly overexpressed in keratinocytes, while levels are low in normal epidermis (53, 54). Moreover, S100A8/A9 has been identified as a differentiation marker of mammary epithelial cells suppressing malignant transition (55).

In healthy neonates, the concentration of S100A8/A9 in the serum is massively increased in the first days of life up to mean levels of >3,000 ng/ml that in adults are only detectable in inflammatory diseases (27). The high serum levels of S100A8/A9 after birth rapidly decrease but reach normal adult levels (≤ 330 ng/ml) not before the second week of life. Hitherto, two sources have been identified that contribute to the high serum levels of S100A8/A9 in newborn infants. Firstly, neonatal blood monocytes and macrophages overexpress and strongly release S100A8/A9, while expression in the corresponding cell types in healthy adult individuals is low (8, 27, 45). Neonatal granulocytes probably also express S100A8 and S100A9 at high levels, but this still needs to be demonstrated. Secondly, human breast milk contains the highest amounts of S100A8/A9 hitherto found under physiologic conditions (56). In healthy term newborns, the levels in breast milk are in average at least six times higher than the already elevated S100A8/A9 serum levels. The kinetics of S100A8/A9 breast milk levels resemble much that in the neonate's serum, i.e., levels are highest in the colostrum and then gradually decrease over time, reaching normal adult serum levels 1 month after birth. In exclusively breast milk-fed human infants, fecal levels of S100A8/A9 were shown to be significantly higher than in formula-fed ones (57). In mice, enterally supplied S100A8/A9 was demonstrated to be systemically bioavailable in the blood circulation (56). In humans, the dependence of the height of S100A8/A9 levels in the serum on the supply by breast milk awaits quantification. Maternal myeloid cells certainly represent a major production site of the high amounts of S100A8/A9 in human breast milk (58) as levels are three times higher if breast milk samples are analyzed without prior depletion of cells (unpublished data of the authors). To what extent mammary epithelial cells (55) increase the production of S100A8/A9 in the lactating human breast and release it into the milk is currently unknown.

A remarkable aspect related to the physiologically high levels of S100A8/A9 in newborn infants is its unsuitability as an inflammatory biomarker, underlining once more the importance and different role of S100A8/A9 in the neonatal period as compared to later childhood and adulthood. In children and adults, the serum level of S100A8/A9 is clinically used as an excellent biomarker in inflammatory processes like sepsis (42, 43), rheumatoid arthritis, juvenile idiopathic arthritis, and autoinflammatory diseases (40, 59–62). In contrast, in neonates, attempts to establish S100A8/A9 as a clinical biomarker of neonatal sepsis remained without tangible success. Two groups proposed serum S100A8/A9 as a promising sepsis marker in very low birth weight infants (63) respective of infants of all gestational ages (64). Both groups surprisingly claimed that S100A8/A9 values were not influenced by postnatal age and the area under receiver operating characteristic (ROC) curve of 0.6 at a cutoff level of 2,200 ng/ml (64) unveiled S100A8/A9 as a rather poor discriminator.

The release of S100A8/A9 from myeloid cells is a specific and energy-dependent process (65). Any sort of stress and cell damage triggers the release of S100A8/A9 like infections, malignancies, burns, and trauma (22, 27, 29, 37–39, 41, 51, 54, 59–62, 66, 67). The interaction of activated endothelium with phagocytes was also described as an important stimulus for S100A8/A9 secretion (66). A study in marathon runners was the first one revealing that heavy exercise represents a physiologic stress trigger leading to increased S100A8/A9 serum levels during the early post-exercise period that returned to normal levels 1 day after the run (68). Labor and birth are certainly one of the most exhausting conditions in life, imposing heavy stress on the mother as well as the newborn infant. Thus, birth-related stress is probably the main trigger of perinatal S100A8/A9 release, and the gradual resolution of stress after birth well explains the postnatal decrease of S100A8/A9 in breast milk (56) and the infant's blood circulation (27). Therewith in line is a previous report that glucocorticoids can induce the expression of S100-alarmins (69). S100A8-producing macrophages were significantly elevated in rheumatoid arthritis patients treated with high-dose steroids. Further trigger for the release of S100A8/A9 in the perinatal context are thinkable but would need validation, e.g., the hormonal changes upon birth giving like the strong increase of estrogens and oxytocin or the withdrawal of progesterone (70).

The major determinant of birth-induced stress is the extent of labor and uterine contractions which is absent in elective cesarean section (CS) (71). This explains why S100A8/A9 levels in breast milk were significantly higher after vaginal delivery (VD) compared to delivery by CS (56). Moreover, the gestational age has an influence on the height of S100A8/A9 levels. S100A8/A9 levels in breast milk of mothers who gave birth to term babies were significantly higher than of mothers with premature born infants (56). The same holds true for S100A8/A9 levels in cord blood of preterm infants compared to term ones (8). Various other parameters like birth weight, gender, Apgar score, hormonal status of the neonate and the mother, maternal body mass index, and perinatal medications might also influence the early-life S100A8/A9 levels and are currently analyzed in the frame of a prospective multicenter clinical study (BMBF 01GL1746B, DRKS00013197) (72).

Summarized, clearly identified states of early-life S100A8/A9 deficiency are premature birth, elective CS, and formula feeding. The manifold reported associations of all these conditions with an unfavorable short-term as well as long-term immune adaptation including an increased susceptibility to infections (5, 9, 10, 14) and a higher risk for developing chronic inflammatory diseases (2, 3, 11, 12, 14) are a strong indicator for S100-alarmins being a possible common molecular denominator setting the stage for successful postnatal immune adaptation.

## Role of S100A8/A9 for the Postnatal Adaptation of Systemic Immunity

**Figure 2:**
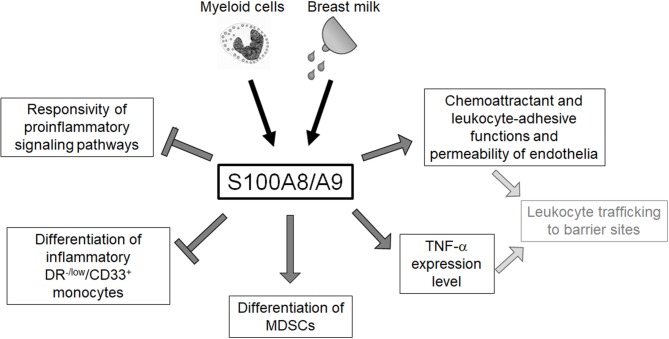
Overview of the regulatory impact of S100A8/A9 on the neonatal innate immune system. Neonatal blood monocytes and breast milk are sources of S100A8/A9 that contribute to the high serum levels of these alarmins in newborn infants. S100A8/A9 constrains systemic inflammatory responses of neonates by tolerizing innate signaling pathways and regulating the expansion of inflammatory and anti-inflammatory myeloid subpopulations. S100A8/A9 impacts directly and indirectly on the leukocyte-recruiting functions of the vascular endothelium S100A8/A9 which suggests an important role in warranting the essential postnatal trafficking of leukocytes to barrier sites.

The neonatal immune system has traditionally been regarded as “deficient” and the high susceptibility to infections has been understood as a “general weakness.” This concept referred to numerous experimental studies that found impaired inflammatory responses of neonatal immune cells to different microbial challenges (5, 13, 14, 73). The expansion of certain suppressive cell types, e.g., regulatory T cells (Tregs) and granulocytic myeloid suppressor cells (MDSCs) observed in neonates was interpreted as part of this concept (74, 75). However, the hallmark of neonatal sepsis is an extremely rapid course with a hyperinflammatory immune response (76), and the inconsistency of experimental and clinical findings remained unsolved for a long time. The disclosure of diverse molecular and cellular effects of physiologically high amounts of S100-alarmins on the neonatal immune system (Figure 2) significantly contributed to the change of concept that the previously misunderstood characteristics of neonatal immunity actually represent an essential programming that warrants postnatal immune adaptation.

**Figure 3:**
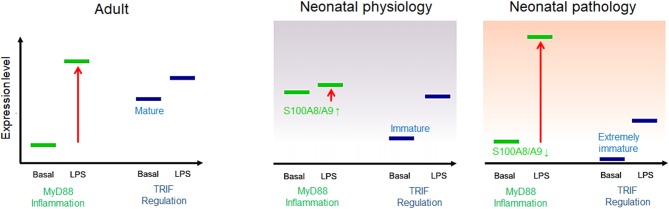
Model of adult and neonatal regulation of lipopolysaccharide (LPS)-responsive gene expression. In healthy adults, MyD88-dependent pro-inflammatory genes are not expressed at baseline but strongly induced by LPS; the basal expression tonus of regulatory TIR domain-containing adaptor protein-inducing interferon (IFN)-β (TRIF)-dependent genes in adults is high and moderately increased by LPS stimulation. In healthy neonates, MyD88-dependent gene expression is physiologically pre-activated by S100A8/A9 and thereby tolerized toward subsequent LPS activation. TRIF-dependent genes are barely expressed at baseline but initiated by a first LPS challenge after birth. In S100A8/A9-deficient neonates, insufficient tolerization of the MyD88-dependent pro-inflammatory response and/or an immature expression tonus or altered postnatal initiation of TRIF-dependent regulatory gene programs results in unregulated hyperinflammatory LPS responses, increasing the risk of severe sepsis.

First of all, it was recognized that the high release and supply of S100A8/A9 directly at the beginning of life corresponds to a conditioning of the neonatal immune system with S100-alarmins and that such pretreatment with S100-alarmins results in stress tolerization of immune cells toward subsequent microbial stimuli (27). The importance and biological relevance of this specific mechanism in newborn infants was further elucidated by Ulas et al. (8) who could link the impaired response of neonatal monocytes to LPS to an activated initial state of MyD88-dependent gene expression at baseline. They further identified S100A8/A9 as an inducer of this specific activation state of neonatal monocytes that was transcriptionally as well as epigenetically fixed as long as serum levels of S100A8/A9 were high. Withdrawal or blocking of S100A8/A9 terminated the activation of the MyD88-dependent gene program and came along with an increased inflammatory response toward microbial stimuli *in vitro* and *in vivo* in experimental sepsis models (8, 27, 45). Furthermore, S100A8/A9 also caused a metabolic programming in cord blood macrophages that was characterized by an impaired glycolysis pathway and suppressed mammalian target of rapamycin (mTOR) activation linked to the inflammatory hyporesponsiveness (77). Importantly, S100A8/A9 regulated the inflammatory responsivity without impairing the antimicrobial functions of neonatal phagocytes (8), a decisive requirement to achieve the critical balance between unimpaired defense and uneventful tolerance development. Consequently, the pretreatment of *S100a9*-knockout mice with the S100A8/A9 heterodimer or the more potent S100A8 homodimer directly after birth rescued murine neonates from fatal courses of later sepsis (8, 45). In human neonates, S100A8/A9 serum levels were negatively associated with the risk of sepsis and significantly higher in term compared to preterm infants. Additionally, in preterm infants, levels higher than 2,000 ng/ml in cord blood were associated with a 25-fold lower risk of late-onset sepsis compared with levels <330 ng/ml (8). This finding closed the gap regarding the seeming discrepancy between the *in vitro* inflammatory hyporesponsiveness of S100-conditioned neonatal immune cells and the clinical characteristic of hyperdynamic neonatal sepsis with overshooting inflammatory responses in S100-deficient states (Figure 3).

This relationship became even more important when evidence was provided that TRIF-dependent regulatory gene programs including IFN-response genes are low expressed and epigenetically silent in neonatal monocytes (8). Their expression tonus was not affected by S100A8/A9 and only gradually increased over a prolonged period of time during the first year of life, which suggests environment-dependent initiation. In parallel, another group demonstrated that preterm infants specifically differ from term infants by an even lower baseline expression of regulatory IFN-response genes (78). The profound immaturity of cell-autonomous regulation together with the impaired age-specific alternative kind of regulation by S100A8/A9 provided an explanation for the massively increased sepsis risk of individual preterm infants (Figure 3). Moreover, identifying this period as a critical phase in which the regulation of inflammatory responsiveness shifts from S100A8/A9-programming to an adult-like endogenous regulation allowed understanding why the incidence of late-onset sepsis is highest in the first 2 weeks of life (79).

Next to the outlined molecular effects of S100 programming, S100A8/A9 has influence on the differentiation of several cell types of the innate immune system. In humans and mice, high levels of S100A8/A9 prevented the expansion a specific subpopulation of inflammatory blood monocytes in neonates (DR^−/low^/CD33^+^ monocytes in humans and CD11b^+^/Gr-1^int^/Ly6G^−^/Ly6C^hi^ cells in mice) which promoted systemic hyperinflammatory responses (45). Treating *S100a9*-knockout neonates directly after birth with S100A8/A9 prevented excessive expansion of this inflammatory monocyte population and death from septic shock. Among the rapidly growing list of mechanisms controlling overshooting inflammation in newborn infants are granulocytic MDSCs. Similar as S100A8/A9 levels, MDSCs are expanded in breast milk and the blood circulation of newborn infants and drop down to low adult-like levels 1 month after birth (75, 80, 81). Initially considered as a sign of immune immaturity, their suppressive activity on the inflammatory phenotype of neonatal T cells and monocytes has meanwhile been included into the concept of alternative immune regulation during the neonatal period (81–83). S100A8 and S100A9 are two of the most important positive regulators of the number and function of MDSCs by acting on hematopoietic progenitor cells (84–88). It was recently shown that S100A8/A9 triggers the suppressive activity and also the antibacterial activity of neonatal MDSCs, thereby controlling inflammation (82). Another important process in the neonatal period is the exchange of fetal tissue-resident leukocytes by hematopoietic blood-derived leukocytes, which is an integral part of immune adaptation and results in the reorganization of the leukocyte profiles at barrier sites (89–92). In this context, the impact of S100A8/A9 on the vascular endothelium as well as on monocytes/macrophages might be of biological relevance. S100A8/A9 induces a specific endothelial response characterized by the induction of chemokines and adhesion molecules and a loss of cell–cell contacts that increases the vascular permeability and promotes leukocyte recruitment (67). Furthermore, TNF-α has been demonstrated to be a pivotal mediator maintaining the postnatal trafficking of leukocyte to barrier sites in the neonatal period (93). By increasing the expression of TNF-α in neonatal monocytes/macrophages (8) together with its impact on the chemoattractant and leukocyte-adhesive function and the permeability of endothelia S100A8/A9 might be crucially involved in orchestrating the extravasation and redistribution of leukocytes in the neonatal period.

Finally, it should be noted that glucocorticoids induce the expression and release of S100-alarmins (69). Antenatal corticosteroids for women at risk of imminent preterm birth are the major perinatal intervention to reduce the incidence of respiratory distress syndrome and neonatal mortality associated with preterm birth (94). The effects of steroid-induced S100-alarmins on neonatal immunity are probably part of the beneficial impact of antenatal corticosteroids in promoting fetal maturation and improving postnatal adaptation.

## Concluding Remarks

A large body of evidence suggests that S100A8/A9 programs the inflammatory responsivity of systemic innate immunity of newborn infants at multiple molecular and cellular levels. Current data suggest that the tuning of the initial programming of immunity by S100-alarmins is an important determinant of how newborn infants react toward the new antigenic challenges in the extrauterine world. Deficient priming by S100-alarmins increases the risk of life-threatening systemic inflammatory response syndromes, which hampers uneventful immune adaptation and regulated reprogramming of immunity by the environment. Better understanding of how the host contributes to the postnatal development of immunity is an opportunity to exploit endogenous mechanisms like S100-alarmins for intervention strategies, which ensure favorable immune adaptation and could benefit health not only in infancy but also in adulthood.

## Author Contributions

DV conceptualized and composed the manuscript.

## Conflict of Interest

The author declares that the research was conducted in the absence of any commercial or financial relationships that could be construed as a potential conflict of interest.
